# Yinma Jiedu granules combined with antibacterial drugs for patients with community-acquired pneumonia: protocol for a multicenter, randomized, double-blind, placebo-parallel controlled clinical trial

**DOI:** 10.3389/fmed.2025.1612945

**Published:** 2025-09-09

**Authors:** Yuewei Li, Jingmin Xiao, Feiting Fan, Luoqi Lin, Yihe Chi, Xinyuan Tan, Wenhan Zhao, Weiliang Wang, Lizhi Zhou, Yinshu Cao, Lei Wu, Lin Lin, Yuanbin Chen

**Affiliations:** ^1^State Key Laboratory of Traditional Chinese Medicine Syndrome, The Second Clinical College of Guangzhou University of Chinese Medicine, Guangzhou, China; ^2^The Second Affiliated Hospital of Guangzhou University of Chinese Medicine/Guangdong Provincial Hospital of Chinese Medicine, Guangzhou, China; ^3^Clinical Trial Service (Guangzhou) Co., Ltd., Guangzhou, China; ^4^Guangdong Provincial Key Laboratory of Clinical Research on Traditional Chinese Medicine Syndrome, Guangzhou, China

**Keywords:** community-acquired pneumonia, randomized controlled trial, Yinma Jiedu granules, Chinese patent medicine, clinical symptoms

## Abstract

**Background:**

Community-acquired pneumonia (CAP) is a severe respiratory infection that can lead to a range of health issues. Yinma Jiedu (YMJD) granules, a representative classic traditional Chinese patent medicine, are widely used in China, yet high-quality evidence regarding their efficacy in alleviating clinical symptoms remains limited. This study aims to evaluate the efficacy and safety of YMJD granules for the treatment of CAP.

**Methods:**

This multicenter, randomized, double-blind, placebo-controlled parallel clinical trial will be conducted at 11 hospitals in China, with each center enrolling patients competitively. A total of 226 CAP patients who meet the inclusion criteria will be stratified by age and then randomized in a 1:1 ratio to the intervention group (*n* = 113; YMJD granules) or control group (*n* = 113; YMJD placebo). All patients will receive a 7-day treatment course followed by a 7-day follow-up for assessment. The primary outcome is the clinical symptom improvement rate on day 4. Secondary outcomes include the clinical cure rate, the improvement rate of individual symptoms, the Cough and Sputum Assessment Questionnaire, the improved pneumonia imaging absorption evaluation scale, the CURB-65 score, the dosage and course of antibiotics, the changes in inflammatory markers, Traditional Chinese Medicine (TCM) syndrome scores and the amount of emergency drug used. Adverse events will also be assessed. Subgroup analyses will explore heterogeneity in YMJD treatment effects.

**Discussion:**

This trial is the first large-sample, randomized, double-blinded, placebo-controlled study of YMJD granules in patients for CAP. It aims to elucidate the mechanisms of early symptoms improvement, identify optimal patient populations, and informing new therapeutic strategies.

**Ethics and dissemination:**

This study design was approved by the Ethics Committee of Guangdong Provincial Hospital of Chinese Medicine (Reference Number: BF2022-306-01).

**Clinical trial registration:**

https://www.chictr.org.cn/showproj.html?proj=189230, Identifier ChiCTR2300072343.

## 1 Introduction

Community-acquired pneumonia (CAP) is one of the primary causes of hospitalization and death from infectious diseases worldwide (1), characterized by lung infiltrates on imaging accompanied by clinical manifestations including fever, dyspnea, and productive cough (2). In China, CAP demonstrates an incidence of 7.13 per 1,000 person-years (3), with median hospitalization costs reaching $556.50 for low-risk cases (4), collectively imposing a substantial health and economic burden. The core principle of CAP treatment is anti-infection, respiratory fluoroquinolones or *β*-lactam/macrolide combination therapy are recommended as first-line regimens (5). However, antibiotic overuse raises concerns about complications, including clostridium difficile-associated diarrhea, gastrointestinal disturbances, and hepatorenal toxicity (6–8), where prolonged regimens escalate adverse event risks. Emerging evidence suggests that *β*-lactam/macrolide combinations have limited efficacy against atypical pathogens in outpatient settings (9, 10), highlighting the need for precision in early therapeutic interventions. Therefore, how to alleviate clinical symptoms and reduce antibiotic use in the early stages of CAP has become the key problem in treatment.

Although Traditional Chinese Medicine (TCM) does not utilize the specific disease name CAP, it categorizes this condition based on its clinical manifestations within the scope of the “cough disease.” According to the Guidelines for Traditional Chinese Medicine Diagnosis and Treatment of Community Acquired Pneumonia (2018 Revision) (11), the pathogenesis of CAP is primarily attributed to two key aspects: (1) exogenous pathogen invasion compromising the lung’s defensive system, or (2) vital qi deficiency impairing resistance against pathogenic factors. The core therapeutic principle focuses on eliminating pathogens and reinforcing vital qi. Emerging evidence supports TCM as a promising adjunct therapy for CAP. Its treatment mechanism include microbial invasion (bacterial, viral, etc.), excessive inflammatory response mediated and oxidative tissue damage (12, 13). The YMJD formula, developed based on the TCM concept of “lung-gut axis coordination,” demonstrates multi-targeted therapeutic effects against these pathological processes. Previous randomized controlled trials (RCTs) demonstrate YMJD’s ability to improve acute bronchitis through inhibitition of inflammatory factors including IL-1β and TNF-*α*, along with improvements in bowel movements and pulmonary function (14, 15). Evidence from experiments has also shown that the components of YMJD granules can reduce the levels of the infiltration of white blood cells, lymphocyte, mononuclear cells and neutrophils, with the inhibition of granulation tissue proliferation in mice (16–18), especially for *Staphylococcus aureus* (19), one of the main pathogen in CAP. Hence, the YMJD granule could potentially serve as a traditional Chinese medicine formula for treating CAP.

Currently, there is a lack of large-scale randomized controlled trials evaluating YMJD granules for CAP under evidence-based principles, particularly regarding early symptom improvement. To address this gap, we will conduct a prospective, multicenter, randomized controlled trial to assess the clinical benefits of combining YMJD granules with standard antibiotics within the critical first 72 h after symptom onset. This study aims to determine the efficacy and safety of YMJD granules in CAP patients while providing high-quality evidence for integrating TCM into CAP treatment strategies.

## 2 Methods

### 2.1 Study design

**Figure 1:**
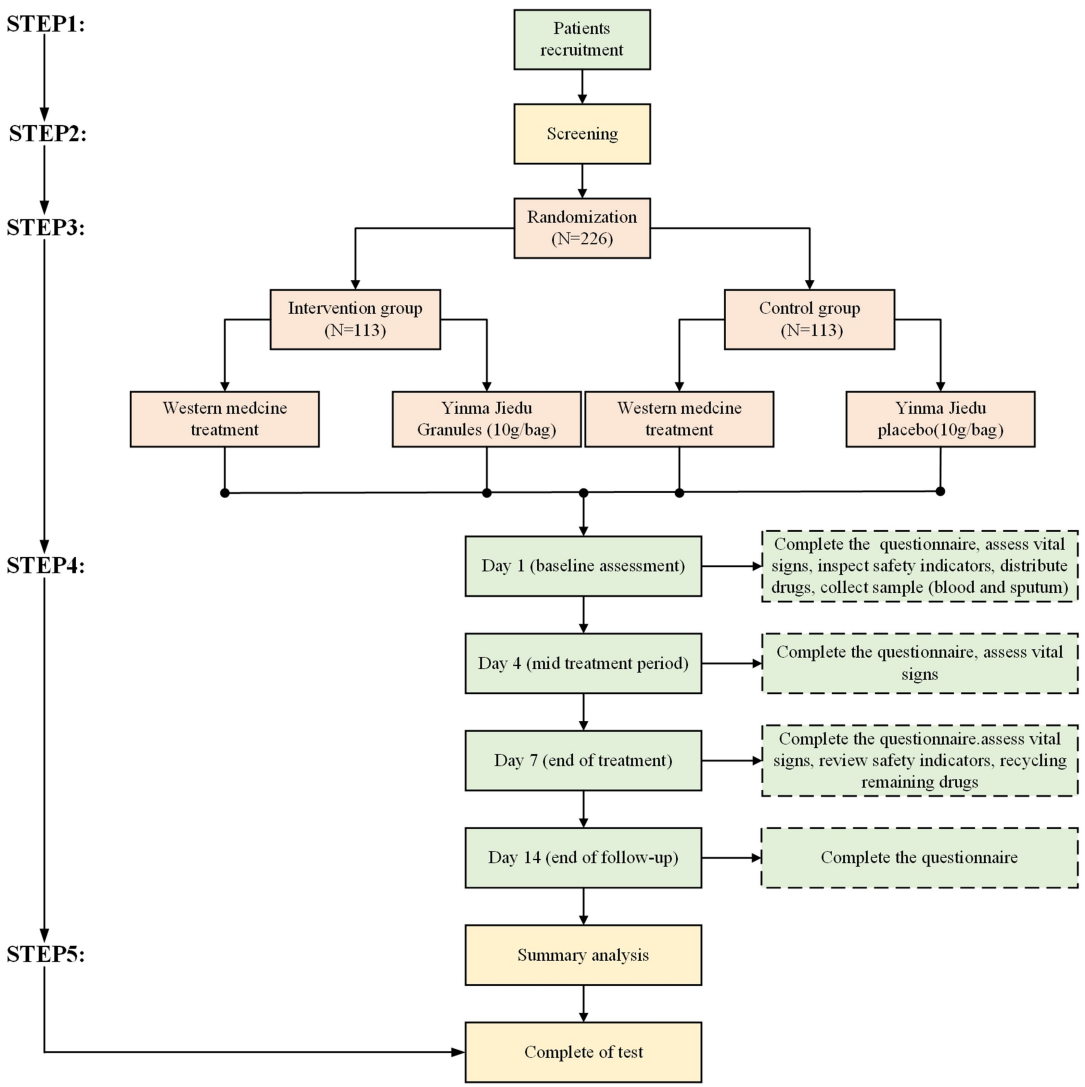
The flow diagram of the trial.

This is a randomized, double-blind, placebo-controlled, multicenter study involving 11 hospitals in China. The study flow is designed strictly according to the Consolidated Standards of Reporting Trials (CONSORT) and Standard Protocol Items: Recommendations for Interventional Trials (SPIRIT). It aims to evaluate the safety and efficacy of YMJD granules combined with antibacterial drugs for CAP. Patients and the public were not involved in the design, conduct, reporting, or dissemination plans of our research. The overall flow diagram of the research procedure is shown in Figure 1.

### 2.2 Study population

#### 2.2.1 Participant recruitment

A total of 226 patients with CAP who meet the eligibility criteria, will be recruited from 11 centers participating in this study. These include one main center, the Guangdong Provincial Hospital of Chinese Medicine, and 10 cooperating centers: Guangdong Second Traditional Chinese Medicine Hospital; The First Affiliated Hospital in Guangzhou University of Chinese Medicine; The Second Affiliated Hospital of Tianjin University of Traditional Chinese Medicine; Shenzhen Traditional Chinese Medicine Hospital; Foshan Traditional Chinese Medicine Hospital; The First Affiliated Hospital of Henan University of Chinese Medicine; The Affiliated Hospital of Jiangxi University of Traditional Chinese Medicine; Guangzhou Panyu District Traditional Chinese Medicine Hospital; Guangdong Qifu Hospital and Huizhou Third People’s Hospital. At each center, a trained study coordinator will identify potentially eligible patients and work with the attending physician to determine their suitability for the trial. Once the patient is confirmed to meet the inclusion and exclusion criteria, they will be enrolled in the trial. Enrolled began in January 2023 and will conclude by December 2027.

#### 2.2.2 Inclusion criteria

Each patient had to satisfy the following inclusion criteria before entry into the study:

1. Patients are between 18 and 80 years of age.
2. Meeting the diagnostic criteria for CAP in the “Guideline of Diagnosis and Treatment for Community-Acquired Pneumonia among Adults in China (released in 2016) (20)”.
3. Meeting the “Guidelines for Traditional Chinese Medicine Diagnosis and Treatment of Community Acquired Pneumonia (2018 Revision) (11) for the “*syndrome of wind-heat invading lung*” or “*syndrome of phlegm-heat obstructing lung*” in Chinese medicine (21).
4. The clinical symptoms should manifest as at least 2 of the following 4 main symptoms: cough, expectoration, dyspnea (shortness of breath), and pleurisy chest pain.
5. Patients with CURB-65 score 0–2 (22).
6. Before recruitment, clear notification and signing of informed consent are required.

#### 2.2.3 Exclusion criteria

1. Severe CAP patients.
2. Interstitial pneumonia, viral pneumonia, and hospital-acquired pneumonia (including ventilator-associated pneumonia).
3. Patients with serious complications such as lung abscess, atelectasis and empyema.
4. Patients complicated with other serious respiratory diseases, such as chronic obstructive pulmonary disease, bronchiectasis, and active tuberculosis.
5. Patients combined with severe brain, heart, liver, renal, and blood system diseases, as well as mental illness.
6. Use of Chinese herbal medicine or Chinese patent medicine that have effects of clearing heat and reducing phlegm and relieving cough or had the antibacterial effect clearly defined by modern pharmacology within 48 h prior to enrollment.
7. Liver function (ALT, AST) or kidney function (serum creatinine) exceeds 2 times the upper limit of normal value.
8. One of the following three conditions (23): ① fullness or hidden pain in the stomach and epigastrium, the stomach likes to press and warm, loose stools, weakness, pale tongue with teeth marks on the sides; ② fullness in the stomach and epigastrium, aggravated by cold, noisy acid reflux, dry mouth and bitterness, cold limbs, and loose stools; ③ recurrent loose stools or diarrhea, which may occur or aggravate at the slightest carelessness in diet, pale tongue.
9. Patients with diseases that severely affect the immune system such as malignancy, organ or bone marrow transplantation, HIV infection, or patients taking immunosuppressive drugs or systemic glucocorticoids within the last 3 months.
10. Patients with dysphagia or a history of gastrointestinal disorders that interfere with the absorption of the test drug (including but not limited to chronic diarrhea, reflux esophagitis, ulcerative colitis, intestinal tuberculosis, gastrinoma, short bowel syndrome, post major gastrectomy, etc.).
11. Combined with diabetes and unstable glycemic control before this episode, glycated hemoglobin control target >7%.
12. Previous history of drug addiction or drug abuse.
13. Pregnancy, planned pregnancy or lactation women.
14. Previous history of severe allergic reactions or known history of hypersensitivity to YMJD granules or placebo ingredients.
15. Patients who have participated or are participating in other clinical trials within the last 3 months.
16. Patients who, in the opinion of the investigator, are poorly adherent and have difficulty completing or complying with the requirements of this trial.

#### 2.2.4 Withdrawal criteria and management

Patients will be required to withdraw for the following reasons.

1. Patients’s condition worsens, requiring discontinuation of the trial.
2. Serious complications, allergic reactions, significant physiological changes that occur during the trial.
3. Using other treatment methods or drugs that are prohibited from being used in combination.
4. Serious adverse events during the study period.
5. Patients are unwilling to continue the trial and ask to withdraw from the trial.
6. Poor subject compliance of patients (such as taking drugs in violation of the prescribed dose, or using prohibited drugs).
7. Poor compliance of patients (trial medication adherence <80% or >120%).
8. Blind release or emergency unblinding.
9. Researchers decided to withdraw the patient from the trial.

### 2.3 Allocation and blinding

A central Interactive Web Response System (IWRS) will assign unique treatment codes for each patient, as well as bottle numbers and instructions for dispensing the blinded study drug that matches the assigned treatment. This study will use the Electronic Data Capture System (EDC) (Copyright^©^ 2012–2023 Clt. Inc. V1.0) to collect research data, which is managed by the Clinical Trial Service (Guangzhou) Co., Ltd. According to the research plan, an electronic Case Report Form (eCRF) will be designed and an EDC database will be established. The database complies with China’s Good Clinical Practice (GCP) guidelines. We will use stratified randomization, with age as the stratification factor. Each center will compete for enrollment, and eligible cases will be allocated to the YMJD granules treatment group or YMJD placebo control group in a 1:1 ratio through the central random allocation system.

This double-blind trial maintains blinding integrity through independent unblinded personnel according to the double-blind trial standards and procedures to ensure the blind status of the trial group by the sponsor, researcher, relevant researchers, and patients. The unblinded personnel will include pharmacists responsible for the preparation and dispensing of the investigational drug and placebo, an independent statistician who will generate the randomization sequence and manage blinding code documents, and a monitor external to the research team who will conduct periodic audits of drug distribution records and blinding compliance to ensure protocol adherence. Following trial completion, patients and investigators will complete a blinding assessment questionnaire to guess group allocation (intervention or placebo).

The study employs a central system for emergency unblinding without paper correspondence. Unblinding is reserved for urgent medical needs or regulatory requests. Before unblinding, the sponsor must be notified, and the principal investigator will execute the process, noting the reasons. The unblinding details and any adverse events are meticulously recorded.

### 2.4 Interventions

After being recruited, patients will be stratified by age and then randomly allocated to either the intervention group or the control group (in a 1:1 ratio). Both groups can be treated with conventional Western medicine, according to the “Guideline of Diagnosis and Treatment for Community-Acquired Pneumonia among Adults in China (released in 2016) (20),” which sets the following criteria for routine use of antimicrobial drugs in hospitalized CAP patients: intravenous cephalosporin; intravenous cephalosporin combined with macrolides; intravenous quinolones. In addition, oxygen therapy, antipyretics, and rehydration support are used as needed. Based on the treatment of conventional Western medicine, patients will receive YMJD granules or YMJD placebo three times per day for 7 days, taking one bag after each meal. The YMJD placebo is consistent with YMJD granules in terms of packaging, appearance, odor, and taste, the composition is shown in Table 1. The drug quality standard complies with the provision of the Chinese Pharmacopoeia and GMP.

**Table 1:** Drug composition.

Drug	Specifications	Producer	Composition
Yinma Jiedu granules	10 g/bag	Yangtze River Pharmaceutical Group Sichuan Hairong Pharmaceutical Co., Ltd., China	Honeysuckle flower, purslane herb, plantain herb, rhubarb root and rhizome, and licorice root
Yinma Jiedu placebo	10 g/bag	Yangtze River Pharmaceutical Group Sichuan Hairong Pharmaceutical Co., Ltd., China	Dextrin, caramel coloring, fructokinin, sucrose octaacetate, sucralose, PVP K30, DL-Tartaric acid, hawthorn flavor, pickled plum, flavoring, anhydrous ethanol and purified water

Except for the experimental drugs, patients are not permitted to use any therapeutic agents that may affect the determination of efficacy, such as cough suppressants and phlegm reducers, glucocorticoids. In addition, the utilization of Chinese patent medicines, Chinese medicines, Chinese medicine granules, or TCM characteristic therapy aimed at clearing heat, resolving phlegm and relieving cough are also prohibited during the trial. If any other medication or therapy is required to treat concomitant diseases, the name of the drug or therapy, actual dosage, dosing frequency, and start/stop time will be well-documented.

### 2.5 Outcomes

#### 2.5.1 Primary outcome

##### 2.5.1.1 Clinical symptom improvement rate on day 4

The primary outcome is early clinical response, defined as an improvement in at least two of four symptoms (cough, chest pain, sputum production, dyspnea) without any exacerbation of any symptom within 96 h following the initial dose of study medication in the intention-to-treat population (24).

An improvement in symptoms is defined as a reduction in severity by a minimum of one grading level. Refer to Table 2 for details.

**Table 2:** Community-acquired pneumonia symptom severity assessment scale.

Symptoms	Asymptomatic	Mild	Moderate	Severe
Cough	No cough	Coughing, but does not interfere with daily activities	Frequent cough that interferes with daily activity	Coughing day and night, most daily activities limited, sleep disrupted
Expectoration	No expectoration	Mild expectoration	Moderate expectoration	Excessive expectoration
Pleurisy pain	No pneumonia-related chest pain	Occasional chest pain during deep breathing, but does not interfere with daily activities	Chest pain during normal breathing, affecting daily activities	Chest pain occurs during rest or shallow breathing, and most daily activities are limited
Dyspnea	No dyspnea	Shortness of breath during intense activities, but does not interfere with daily activities	Frequent dyspnea that interferes with daily activity	Dyspnea occurs during mild activity or at rest, and most daily activities are limited

#### 2.5.2 Secondary outcomes

##### 2.5.2.1 Clinical cure rate

The clinical cure rate (25) is defined as the proportion of patients in each group with complete resolution of the four major clinical symptoms (cough, sputum, pleuritic chest pain, dyspnea) and no requirement for further antimicrobial therapy by day 14. The clinical cure rate will be evaluated on day 14 of enrollment.

##### 2.5.2.2 Improvement rate of single symptoms (including cough, expectoration, pleurisy chest pain, and dyspnea)

Assess the variations in single clinical symptoms (including cough, expectoration, pleurisy chest pain, and dyspnea) of the patients in the two groups before and after treatment. Symptoms severity is classified into four levels, including asymptomatic, mild, moderate, and severe. Symptom improvement is defined as a decrease in severity by at least one level (e.g., from moderate to mild). The improvement rate of single symptoms will be evaluated on day 1, day 7, and day 14 of enrollment.

##### 2.5.2.3 Cough and Sputum Assessment Questionnaire (CASA-Q)

Assess the changes in cough and sputum symptoms of two groups of patients before and after treatment using the CASA-Q questionnaire (26). The CASA-Q questionnaire will be conducted on day 1, day 7, and day 14 of enrollment.

##### 2.5.2.4 Pneumonia Imaging Absorption Evaluation Scale

Patients record pneumonia uptake by Pneumonia Chest X-ray Absorption Evaluation Scale on day 1 and day 7 of enrollment (27). Two imaging experts will independently evaluate the scores of the improved version of the Modified Pneumonia Imaging Absorption Evaluation Scale, and then take the average value.

##### 2.5.2.5 CURB-65 score

Patients will be assessed for severity of illness and risk of mortality by the CURB-65 score on day 1 and day 7 of enrollment (28), including state of consciousness, blood urea nitrogen value, respiratory rate, blood pressure, and age. An elevated total score indicates a graver condition.

##### 2.5.2.6 Dosage and course of antibiotics

To assess the total dosage and duration of medication administered from enrollment to the end of follow-up for patients in both groups treated with three anti-infective regimens: cephalosporins, cephalosporins combined with macrolides, and respiratory quinolones. Anti-infective therapy may be discontinued if clinical symptoms improve significantly, including vital signs, eating ability, and psychological state, Continuous treatment time not less than 5 days (11, 20).

##### 2.5.2.7 Changes in inflammatory indicators

Evaluate the improvement of systemic infection indicators in two groups of patients on day 1 and day 7 of enrollment, including C-reactive protein, serum procalcitonin, interleukin 6, serum amyloid A.

##### 2.5.2.8 TCM syndrome points

Total TCM syndrome scores and individual symptom scores (cough, expectoration, pleusity pain, and dyspnea) will be assessed on day 1, day 4, day 7 and day 14 of enrollment. Detailed evaluation criteria are summarized in Table 3 (29). TCM syndromes were independently assessed by two physicians, if there were any discrepancies in the determination of TCM syndrome patterns, a third physician would adjudicate the case to ensure the accuracy of syndrome differentiation and minimize bias.

**Table 3:** TCM syndrome points.

Main symptom points	Score			
Fever	□ None (0)	□ Mild (3)	□ Moderate (6)	□ Severe (9)
Cough	□ None (0)	□ Mild (3)	□ Moderate (6)	□ Severe (9)

Secondary symptom points	Score	
Chest and hypochondriac fullness causing pain during coughing	□ No (0)	□ Yes (1)
Yellowish white sputum	□ No (0)	□ Yes (1)
Sweating	□ No (0)	□ Yes (1)
Desire to drink water	□ No (0)	□ Yes (1)
Red face	□ No (0)	□ Yes (1)
Insufficient appetite	□ No (0)	□ Yes (1)
Yellow urine color	□ No (0)	□ Yes (1)
Dry stool	□ No (0)	□ Yes (1)
Abnormal respiratory sounds in the lungs	□ No (0)	□ Yes (1)
Total score	_________	

##### 2.5.2.9 Improvement rate of TCM evidence of efficacy

Improvement rate of TCM syndrome efficacy on day 4, day 7, and day 14 of enrollment. Referring to the criteria for determining the efficacy of the symptoms in the relevant literature, the treatment results were classified into four levels: clinically cured, apparently effective, effective and ineffective (29).

$$\begin{array}{l} \text{Improvement rate of Chinese medical evidence efficacy}=\\ (\begin{array}{l} \text{number of cured cases}+\text{number of apparently}\;\\ \text{effective cases}+\text{number of effective cases}+\\ \text{number of ineffective cases} \end{array})/\\ \text{total number of cases}\times 100\% \end{array}$$

##### 2.5.2.10 The amount of emergency drug used

If patients experience exacerbated symptoms such as coughing and expectoration that they cannot tolerate, the use of Compound Methoxyphenamine Capsules is allowed.

### 2.6 Sample collection

Blood samples (standard EDTA blood collection) will be collected on day 1 and day 7; these will be subsequently centrifuged to separate plasma and serum, and samples will be frozen at −80 °C.

Sputum samples will be collected from each patient sputum induction using hypertonic saline is performed if participant unable to expectoration, based on Chinese national guideline on diagnosis and management of Cough (30).

All of biological samples will be used for muti-omics analysis to explore the biological mechanisms of YMJD.

### 2.7 Safety evaluation

Safety assessments include adverse events, clinical laboratory tests, physical examinations, vital signs, and electrocardiogram (ECG). Vital signs examination, including blood pressure, body temperature, respiration, and pulse, will be collected on the 1, 4 ± 1, and 7 ± 1 days of enrollment. Routine blood and urine tests, liver and kidney function tests and ECGs will be collected on the 1 and 7 ± 1 days of enrollment.

### 2.8 Participant timeline

Patients in the trial are required to be recorded four times, with visits at enrollment, day 4, day 7 and day 14. The specific assessments at each visit are detailed in Table 4.

**Table 4:** Time schedule of enrolment, intervention and outcome measures of the trial.

Study phase	Treatment phase			Follow-up phase
Study day	Day 1	Day 4	Day 7	Day 14
Basic medical history				
Informed consent form	X			
Basic condition	X			
Treatment history	X			
Past medical history	X			
Allergy history	X			
Concomitant medications		X-------------------------------------------X		
Efficacy evaluation				
Chest CT	X		X	
C-reactive protein	X		X	
Serum procalcitonin	X		X	
Interleukin 6	X		X	
Serum amyloid A	X		X	
Efficacy evaluation indicators				
Clinical symptom assessment		X-------------------------------------------X		
Clinical symptom improvement rate on day 4		X		
Improvement rate of single symptoms			X	X
CASA-Q	X		X	X
Improved pneumonia Imaging Absorption Evaluation scale	X		X	
CURB-65 score	X		X	
Dosage and course of antibiotics		X	X	X
Changes in inflammatory indicators	X		X	
TCM Syndrome Points		X------------------------------------------X		
Improvement rate of TCM evidence of efficacy		X	X	X
The amount of emergency drug used		X-----------------------------------------X		
Safety evaluation				
Basic vital signs	X	X	X	
Routine blood test	X		X	
Liver and kidney function tests	X		X	
Routine urine test	X		X	
Urine pregnancy test	If necessary			
ECGs	X		X	
Adverse event assessments		X------------------------------------------X		
Other related work				
Subject screen	X			
Random assignment	X			
Drug distribute	X			
Collect blood and sputum samples	X		X	

### 2.9 Sample size calculation

The sample size calculations for this study was determined based on the primary outcome, which is the improvement rate of clinical symptoms on the 4th day of treatment. We estimated the improvement rate based on literature reviews of published studies (24, 31). The improvement rate was assumed to be 78% for the control group and 92.5% for the treatment group. Statistical tests were performed at a two-sided significance level of 0.05. The test power was set to 1-*β* = 80%, and the sample size ratio of the two groups was 1:1. Pass (version 15.0.5) was used for the analysis. A total of 180 patients are planned to be enrolled in this trial. Considering a dropout rate of about 20%, our target sample size is 226 patients, with 113 in each group.

### 2.10 Statistical analysis

#### 2.10.1 Preset subgroups

We will present subgroups based on the following factors and conduct subgroup analysis on the main efficacy indicators: age, pneumonia severity, TCM syndrome, combined disease, and antibacterial drugs of different classes.

#### 2.10.2 Analysis set

The efficacy will be evaluated by Full Analysis Set (FAS) and Per-protocol Set (PPS), and the safety will be evaluated by Safety Set (SS). The FAS includes data from all randomized patients who received at least one dose of the study drug and had at least one post-randomization assessment. The PPS included all patients who adhered to the trial protocol, had no missing baseline variables, had evaluable primary variables, and had no major protocol violations. The SS included all patients who took at least one dose of the study drug and had safety data recorded.

#### 2.10.3 Analysis method

All analysis and data visualization will be performed using SAS Version 9.4.

For continuous variables, the descriptive statistics will include the count of patients (*n*), along with the mean, median, standard deviation (SD), minimum and maximum values. This model will utilize the changes in indicators from baseline as the dependent variables, while accounting for the treatment groups, random stratification factors, and baseline values as covariates. The least squares mean and the associated 95% confidence intervals will be calculated for the changes from baseline for both the test and control measures, as well as for the differences between the groups, specifically from the test group to the control group.

Categorical data will be presented as frequencies and proportions. The 95% confidence interval for each rate was derived using the Clopper-Pearson method. To assess the differences between the two groups across stratification levels, the Cochran–Mantel–Haenszel (CMH) statistic will be employed, considering the random stratification factors. Furthermore, the stratified Newcombe method, which also accounts for these stratification factors, will be utilized to calculate the rate difference for each indicator from the treatment group compared to the control group, along with its corresponding 95% confidence interval.

#### 2.10.4 Data management

The database builder is responsible for building the EDC database and setting logical validation rules according to the data validation plan. After the database construction is completed, User Acceptance Test (UAT) should be conducted. Only after passing the UAT can the database be officially launched and used. The system administrator creates accounts and assigns different permissions based on different roles such as data entry staff, data managers, investigators (primary), and clinical monitors. During the research process, the investigator or designated data entry staff (clinical coordinators) enter the source data into eCRF promptly and accurately. Original records from the study will be preserved and accessible for this study. The system automatically performs logical validation after data entry and submission, and the data will be reviewed from different perspectives by clinical supervisors, data managers, and medical staff. To ensure the quality of this study, the framework of appropriate planning before the trial, adequate oversight and monitoring, and verification will be implemented.

Upon trial completion, database lock will be implemented after tripartite validation confirms data accuracy. The finalized dataset will be transferred to statisticians designated by the sponsor for analysis. All source documents shall be stored in the EDC, with data extraction privileges restricted exclusively to personnel pre-authorized by the sponsor.

#### 2.10.5 Interim analysis

This trial will establish an independent Data Monitoring Committee (IDMC), consisting of at least two clinical medical experts and one statistical expert who are independent of the trial. The main responsibility of IDMC is to evaluate interim analysis of efficacy and safety data, and review the results based on data analysis. After the first 114 patients (50%) completed the improvement rate of clinical symptoms on day 4 (including dropout and early termination of treatment), the independent statistical team will strictly follow the preset IDMC regulations, conduct unblinded analysis on the data of these 114 patients according to the interim analysis plan and IDMC requirements, and re-estimate the sample size based on the interim analysis results.

## 3 Discussion

Despite increasing evidence confirming the role of drugs in the treatment of CAP, there is a growing clinical concern regarding rapid relief of early-stage symptoms, reduction of mortality rates, and mitigation of antibiotic resistance. As a major component of complementary and alternative medicine, TCM has been applied in CAP for many years (32), some studies have revealed the effect of Chinese patent medicine in treating CAP (33, 34), a kind of herb injection named Xuebijin (35) showed the efficacy in severe CAP and sepsis. This treatment improves pneumonia severity indices and reduces mortality, supported by high-quality evidence. However, previous clinical trials focusing on mild and moderate CAP still have significant limitations. Further research is needed to optimize patient selection criteria, standardize outcome assessments, and thoroughly evaluate the safety of oral Chinese medicine formulations. Therefore, we have designed this multicenter, randomized, placebo-controlled, double-blinded trial to assess the effect and safety of YMJD in CAP, hoping to gain valuable insights related to YMJD that can be implemented as part of the complementary treatment for patients with CAP.

YMJD is a National Technology Product approved by the State Food & Drug Administration (CYZB1303054), authorized by invention patent (CN201410212943.0) and approved for marketing by the State Food & Drug Administration in 2013 (number YBZ00702012). Previous clinical trial demonstrated that the combining YMJD with antibacterial drugs significantly enhanced therapeutic efficacy and alleviated clinical symptoms. The treatment group exhibited showed significantly shorter durations of fever resolution, cough and expectoration disappearance times, rales disappearance time, and hospital stay compared to the control group (*p* < 0.01). Additionally, the treatment group showed superior improvement effects on pulmonary shadows relative to the control group (*p* < 0.05) (36). Furthermore, a destructive phenomenon on the cell walls of *Staphylococcus aureus* was observed in the treatment group (19).

This trial employs an updated central IWRS randomization process to maintain allocation concealment. Investigators and drug administrators will be given independent authority to log into this system after signing a confidentiality agreement. Quality control is vital in multicenter RCTs, so we will use EDC to collect and improve the quality of the data. Instead of finding errors after the data collection period, EDC allows researchers to build logical checks, which can reduce the number of errors in the data at the collection site. Moreover, EDC keeps all traces of operation to ensure the authenticity of data. All data will be managed by independent data management personnel to ensure the authenticity and reliability, which has not been achieved in previous studies. At the same time, we invite independent clinical physicians and radiologists to evaluate TCM syndromes and chest imaging, reducing the subjectivity of researchers and ensuring the reliability of the research results.

Furthermore, we will establish the IDMC, independent of the sponsor, to provide ongoing monitoring of safety data when half of the sample is included in this trial. In strict accordance with the IDMC charter, the data will be analyzed unblinded by an independent statistical team according to the interim analysis plan and the requirements of IDMC, and the sample size will be re-estimated based on the interim analysis results. Besides, the charter for the IDMC will also clearly outline all roles, responsibilities, and decision-making criteria. This approach will enable the IDMC to rigorously safeguard the integrity and quality of the research while minimizing the risk of sample wastage.

The duration of treatment for CAP have not been well established. There is evidence to suggest that treatment durations of 6 days or less may yield different outcomes compared to treatments lasting 7 days or more, with variations in therapeutic effects, mortality rates, and the incidence of severe adverse events (37). Given these considerations, the significance of early and effective intervention cannot be overstated. We choose early clinical response, especially on day 4, as the primary outcome in this trial. This marks the first instance of utilizing this specific outcome in TCM RCTs. This outcome is designed to capture an early improvement of one or more levels, thereby encompassing a multi-dimensional view of patient response. It belongs to a kind of quantitative indicators instead of qualitative ones, and has been used in many famous RCTs such as SOLITAIRE-ORAL (24) and OPTIC (25). More and more investigators choose this scale as the primary outcome to support registration with the FDA in the US. Also, we included other common outcomes to assess the efficacy of YMJD in symptom relief, chest imaging, severity reduction, and Chinese medicine syndrome improvement. We hope to evaluate the efficacy comprehensively and highlight the feature of TCM.

Evidence showed that CAP can be caused by many kinds of microbiota, and the comprehensive understanding of the lung microbiome is essential for the CAP treatment. There is little TCM research for CAP that pays attention to this area. The role of pulmonary microbiota in CAP is currently being researched. Further understanding of the pulmonary microbiota may provide information on the inflammatory response and susceptibility to specific pathogens (38). This study will collect patients’ sputum and blood samples, to explore the lung microbiome before and after YMJD treatment. We hope to provide deeper understanding of the mechanism of Chinese patent medicine in the treatment of CAP.

This study has several limitations. Firstly, the inherent subjectivity of TCM syndrome assessments may introduce bias. Although all investigators received standardized training and utilized unified evaluation criteria, variations in inter-rater reliability could persist across multiple centers during clinical judgment of symptom patterns. Second, despite rigorous matching of the placebo and YMJD granules in appearance, odor, and taste, potential unblinding might occur if participants perceive differential gastrointestinal effects or treatment onset times. We will statistically evaluate blinding success through end-of-trial participant/investigator questionnaires and sensitivity analyses.

To our knowledge, this is the first large-sample, randomized, double-blinded, placebo-controlled trial to investigate the efficacy and safety of YMJD granules among patients with CAP. We anticipate the results to provide insight into the science of Chinese patent medicine for CAP. Understanding how YMJD can improve early clinical symptom, identify advantageous patients and key stages for treatment, which may inform novel therapeutic approaches in the treatment of patients with CAP.

## References

[1] Lozano R Naghavi M Foreman K Lim S Shibuya K Aboyans V . Global and regional mortality from 235 causes of death for 20 age groups in 1990 and 2010: a systematic analysis for the global burden of disease study 2010. Lancet. (2012) 380:2095–128. doi: 10.1016/S0140-6736(12)61728-0, PMID: 23245604 PMC10790329

[2] Metlay JP Waterer GW Long AC Anzueto A Brozek J Crothers K . Diagnosis and treatment of adults with community-acquired pneumonia. An official clinical practice guideline of the American Thoracic Society and Infectious Diseases Society of America. Am J Respir Crit Care Med. (2019) 200:e45–67. doi: 10.1164/rccm.201908-1581ST, PMID: 31573350 PMC6812437

[3] Sun Y Li H Pei Z Wang S Feng J Xu L . Incidence of community-acquired pneumonia in urban China: a national population-based study. Vaccine. (2020) 38:8362–70. doi: 10.1016/j.vaccine.2020.11.004, PMID: 33199077

[4] Song JH Thamlikitkul V Hsueh PR. Clinical and economic burden of community-acquired pneumonia amongst adults in the Asia-Pacific region. Int J Antimicrob Agents. (2011) 38:108–17. doi: 10.1016/j.ijantimicag.2011.02.017, PMID: 21683553

[5] Morrissey I Fernandes P Tacchini C Zampaloni C Hawser S. Activity of solithromycin and comparators against streptococci isolated from respiratory samples collected in 2012–13: 24th European congress of clinical microbiology and infectious diseases. Spain, Barcelona: (2014). 1584 p. Available at: http://www.eucast.org/clinical_breakpoints/

[6] Loo VG Poirier L Miller MA Oughton M Libman MD Michaud S . A predominantly clonal multi-institutional outbreak of *Clostridium difficile*-associated diarrhea with high morbidity and mortality. N Engl J Med. (2005) 353:2442–9. doi: 10.1056/NEJMoa051639, PMID: 16322602

[7] Carmichael H Asch SM Bendavid E. Clostridium difficile and other adverse events from overprescribed antibiotics for acute upper respiratory infection. J Intern Med. (2023) 293:470–80. doi: 10.1111/joim.13597, PMID: 36460621 PMC12129334

[8] Weiss K. Clostridium difficile and fluoroquinolones: is there a link? Int J Antimicrob Agents. (2009) 33:S29–32. doi: 10.1016/S0924-8579(09)70013-5, PMID: 19303566

[9] Postma DF van Werkhoven CH van Elden LJ Thijsen SF Hoepelman AI Kluytmans JA . Antibiotic treatment strategies for community-acquired pneumonia in adults. N Engl J Med. (2015) 372:1312–23. doi: 10.1056/NEJMoa1406330, PMID: 25830421

[10] Mandell LA Waterer GW. Empirical therapy of community-acquired pneumonia: advancing evidence or just more doubt? JAMA. (2015) 314:396–7. doi: 10.1001/jama.2015.3858, PMID: 26219057

[11] Xueqing Y Yang X Jiansheng L. Guidelines for traditional Chinese medicine diagnosis and treatment of community acquired pneumonia (2018 revision). J Tradit Chin Med. (2019) 60:350–60. doi: 10.13288/j.11-2166/r.2019.04.019

[12] Wunderink RG Waterer G. Advances in the causes and management of community acquired pneumonia in adults. BMJ. (2017) 358:j2471. doi: 10.1136/bmj.j2471, PMID: 28694251

[13] Vaughn VM Dickson RP Horowitz JK Flanders SA. Community-acquired pneumonia: a review. JAMA. (2024) 332:1282–95. doi: 10.1001/jama.2024.14796, PMID: 39283629

[14] Wei W Yangqing L Zheng L. Effects of Yinma Jiedu granules on lung function and expression of inflammatory factors in children with acute bronchitis syndrome of phlegm-heat obstructing lung. Med Innov China. (2022) 19:91–5.

[15] Pei-quan C Chun-xin W Yi G. Clinical observation on Yinma Jiedu granule combined with western medicine in treating acute exacerbation of chronic obstructive pulmonary disease. Electron J Clin Med Lit. (2018) 5:16–9. doi: 10.16281/j.cnki.jocml.2018.33.007

[16] Xiao-he L Xi L. Experiment of bacteriostatic activities in vitro in water-decocted liquid of *portulaca oleracea* L.and *houttuynia cordata* thunb. Anti-Infect Pharm. (2010) 7:33–4.

[17] Lan L Rong L. Extract of ingredients for medicine in *lonicera confusa* DC and study on antibiotic activity. Med Sci J Cent South China. (2012) 40:298–300.

[18] Yang K Yangmin M Yanjun L Ning L. Antibacterial activity of extacts from *Plantago asiatica* L. China Brew. (2010) 29:151–3.

[19] Rui Z Yaomei T Xueyan Z Rong H Fengjiao Y Lian L . Antibacterial efftects *in vitro* of Yinma Jiedu granules combined with antibiotics. Chin J Hosp Pharm. (2017) 37:2038–41. doi: 10.13286/j.cnki.chinhosppharmacyj.2017.20.08

[20] Respiratory Disease Branch of Chinese Medical Association. Guideline of diagnosis and treatment for community acquired pneumonia among adults in China (released in 2016). Chin J Tuberc Respir. (2016) 39:253–79. doi: 10.3760/cma.j.issn.1001-0939.2016.04.005

[21] The State Administration for Market Regulatio, Standardization Administration of China. GB/T 16751.2-2021. (2021). Clinic terminology of traditional Chinese medical diagnosis and treatment-Part 2:Syndromes/patterns[S]. 2021-11-26.

[22] Bradley J Sbaih N Chandler TR Furmanek S Ramirez JA Cavallazzi R. Pneumonia severity index and CURB-65 score are good predictors of mortality in hospitalized patients with SARS-CoV-2 community-acquired pneumonia. Chest. (2022) 161:927–36. doi: 10.1016/j.chest.2021.10.031, PMID: 34740594 PMC8562015

[23] Wen YD Lu F Zhao YP Wang P Yang Q Li JX . Epigastric pain syndrome: what can traditional Chinese medicine do? A randomized controlled trial of Biling Weitong granules. World J Gastroenterol. (2020) 26:4170–81. doi: 10.3748/wjg.v26.i28.4170, PMID: 32821078 PMC7403800

[24] Barrera CM Mykietiuk A Metev H Nitu MF Karimjee N Doreski PA . Efficacy and safety of oral solithromycin versus oral moxifloxacin for treatment of community-acquired bacterial pneumonia: a global, double-blind, multicentre, randomised, active-controlled, non-inferiority trial (SOLITAIRE-ORAL). Lancet Infect Dis. (2016) 16:421–30. doi: 10.1016/S1473-3099(16)00017-7, PMID: 26852726

[25] Stets R Popescu M Gonong JR Mitha I Nseir W Madej A . Omadacycline for community-acquired bacterial pneumonia. N Engl J Med. (2019) 380:517–27. doi: 10.1056/NEJMoa1800201, PMID: 30726692

[26] Crawford B Monz B Hohlfeld J Roche N Rubin B Magnussen H . Development and validation of a cough and sputum assessment questionnaire. Respir Med. (2008) 102:1545–55. doi: 10.1016/j.rmed.2008.06.009, PMID: 18662868

[27] Yin R Li M Xu H Gendi J Miao C Lili X . Preliminary development and application of pneumonia chest film absorption evaluation scale. Chin J Respir Crit Care Med. (2012) 11:185–7.

[28] British Thoracic Society: Adult Community Acuired Pneumonia Audit 2018–2019, reports Vol 10, Issue 4, (2019). Available online at: https://www.brit-thoracic.org.uk/quality-improvement/clinical-resources/adult-community-acquired-pneumonia (Accessed Nov, 2019).

[29] Feng J Liu N Yang S L M Zhong X Du Y . A clinical study on the treatment of community acquired pneumonia with the "real world" method of strengthening and detoxifying virtues. Lishizhen Med Mater Med Res. (2020) 31:121–4.

[30] Asthma Group of Chinese Thoracic Society. Chinese national guideline on diagnosis and management of cough (2021). Zhonghua Jie He He Hu Xi Xi Ji Bing Za Zhi. (2022) 45:13–46. 10.3760/cma.j.cn112147-20211101-00759 35000304

[31] Ye S Gong G Zheng H Guohua H Xia T. Cytokine changes in community-acquired pneumonia in elderly and intervention of traditional Chinese medicine. Zhongguo Zhong Yao Za Zhi. (2010) 35:1486–9. 20822027

[32] Suofang S. Application of professor Zhou Zhongying's theory of stasis-heat to pulmonary diseases: insights from clinical evidence. Chin Med Mod Distance Educ China. (2013) 11:113–4.

[33] Fan Y Liu W Wan R Du S Wang A Xie Q . Efficacy and safety of yinqiao powder combined with western medicine in the treatment of pneumonia: a systematic review and meta-analysis. Complement Ther Clin Pract. (2021) 42:101297. doi: 10.1016/j.ctcp.2020.101297, PMID: 33360842 PMC7834461

[34] Li X Wei S Ma X Li H Jing M Liu H . Efficacy and safety of Tanreqing injection combined with antibiotics against *Streptococcus pneumoniae* pneumonia: a systematic review and meta-analysis. J Clin Pharm Ther. (2022) 47:1159–72. doi: 10.1111/jcpt.13706, PMID: 35712904

[35] Song Y Yao C Yao Y Han H Zhao X Yu K . Xue bijing injection versus placebo for critically ill patients with severe community-acquired pneumonia: a randomized controlled trial. Crit Care Med. (2019) 47:e735–43. doi: 10.1097/CCM.0000000000003842, PMID: 31162191 PMC6727951

[36] Zhao W Yan Y Ding X . Clinical observation on 31 cases of community-acquired pneumonia with wind-heat invading lung treated by Yinma Jiedu Keli as adjuvant therapy. J Gansu Univ Chin Med. (2023) 40:60–4. doi: 10.16841/j.issn1003-8450.2023.06.11

[37] Tansarli GS Mylonakis E. Systematic review and meta-analysis of the efficacy of short-course antibiotic treatments for community-acquired pneumonia in adults. Antimicrob Agents Chemother. (2018) 62:e00635–18. doi: 10.1128/AAC.00635-18 29987137 PMC6125522

[38] File TJ Ramirez JA. Community-acquired pneumonia. N Engl J Med. (2023) 389:632–41. doi: 10.1056/NEJMcp2303286 37585629

